# Complex patterns of WNV evolution: a focus on the Western Balkans and Central Europe

**DOI:** 10.3389/fvets.2024.1494746

**Published:** 2024-11-20

**Authors:** Sofija Šolaja, Šejla Goletić, Ljubiša Veljović, Dimitrije Glišić

**Affiliations:** ^1^Department of Virology, Institute of Veterinary Medicine of Serbia, Belgrade, Serbia; ^2^Veterinary Faculty, University of Sarajevo, Sarajevo, Bosnia and Herzegovina

**Keywords:** West Nile virus, phylogenetics, molecular characterization, phylogeography, Serbia

## Abstract

**Introduction:**

West Nile Virus, an emerging zoonotic pathogen, has been circulating in Serbia for over a decade, with its first detection in mosquitoes in 2010. Since then, the virus has led to increasing cases in both animals and humans, peaking in 2018 with 415 human cases and 36 fatalities. This study aimed to explore the phylogenetic relationships between previously sequenced West Nile virus strains from Serbia and those sequenced in this study, while also identifying possible virulence factors.

**Materials and methods:**

Whole genome sequencing was conducted using a targeted approach on the MinION Mk1C platform, following a two-step process involving cDNA synthesis and amplification. Bioinformatics analysis included demultiplexing, primer trimming, and sequence mapping using tools such as iVar, Minimap2, and Samtools. Phylogenetic analysis was performed using MAFFT alignment and the Maximum Likelihood method with the Tamura Nei model in MEGA X software. Virulence factors were assessed in both structural and nonstructural proteins, focusing on key glycosylation motifs and specific mutations. Homology modeling of the E protein was also performed to evaluate potential structural changes due to mutations.

**Results:**

Phylogenetic analysis revealed two major sublineages within the E subclade, representing the majority of strains from Western and Central Europe. These sublineages likely originated from Austria, Serbia, and Hungary between 2008 and 2012. The study also identified three distinct sublineages within the D subclade, which includes more diverse strains from Southern Europe. The E protein exhibited significant variations, particularly at the E159 site, which is crucial for virulence. The EI159T aa change has become dominant in recent years, replacing the previously prevalent EI159M. Additionally, changes in the NS1 glycoprotein and NS3 protein, both of which are involved in immune modulation and viral replication, were identified, with potential implications for the virus’s virulence.

**Conclusion:**

The study’s findings highlight the Western Balkans and Central Europe as key regions for the mixing and dissemination of West Nile virus strains from both Western-Central and Southern Europe. These results underscore the importance of continuous surveillance and phylogenetic analysis to monitor the evolution and spread of West Nile virus, particularly in light of the frequent mutations observed in virulence-associated sites.

## 1 Introduction

West Nile Virus (WNV) is an arbovirus primarily transmitted by mosquitoes to susceptible hosts. It belongs to the species *Orthoflavivirus nilense*, the genus *Orthoflavivirus* within the family *Flaviviridae* (1). While a wide range of hosts can be infected, birds are considered the primary, amplifying hosts. Other vertebrates are regarded as dead-end hosts since they do not contribute significantly to the transmission cycle (2). The viral genome consists of a single-stranded, positive-sense RNA molecule, which encodes one polyprotein. This polyprotein is subsequently cleaved by structural proteases into structural proteins (Capsid (C), Membrane (prM), and Envelope (E)) and nonstructural proteins (NS1, NS2A, NS2B, NS3, NS4A, NS4B, and NS5) (1). The polyprotein is flanked by untranslated regions (UTRs) at both the 3′ and 5′ ends, consisting of approximately 300–600 nucleotides (nt) and 90–92 nt, respectively (2). The prM is a glycoprotein essential for the proper folding and rearrangement of the E protein. Cleavage of prM by furin-like proteases induces global conformational changes, leading to the formation of mature virus particles (2, 3). The E protein is a structural protein composed of 500 amino acids (aa) and is divided into three domains: DI, DII, and DIII. DI, consisting of 127 aa, forms eight-stranded *β*-barrels that comprise the protein’s central region (4). DII, comprising 170 aa, has its proximal end within DI and its distal end forming a fusion loop, which facilitates contact with the host cell (4). The DIII section forms an ectodomain composed of seven *β*-stranded barrels (4). Within the E protein, there is a conserved N-linked glycosylation site that has been associated with the neurovirulence of the virus in vertebrates (2, 4). Certain changes in the E protein have also been linked to increased virulence, such as the EI159V mutation. Under experimental conditions, this mutation showed higher replication in brain tissue compared to the EI159 strain (5). Changes in the NS1 and NS3 proteins, which are involved in immunomodulation of the host response and cleaving junctions in the WNV polyprotein, respectively, have also been associated with increased virulence. A significant number of potential virulence factors have been described for WNV, with different virulence factors depending on the host (2). The virus is genetically unstable, exhibiting high genetic diversity with nine currently described lineages (6).

The earliest description of WNV dates back to 1937 in Uganda, Africa, from where it is believed the virus spread across the continent. In Europe, the earliest recorded instance of WNV was in Albania in 1958, with a resurgence in the 1990s in Romania and Russia, resulting in a high number of human cases (6). These early outbreaks of West Nile Fever (WNF) in the 1990s and early 2000s were primarily connected to WNV lineage 1 (7). An increase in cases occurred in 2004 when a goshawk was found dead in Hungary, and it was later determined that the cause was an infection with WNV lineage 2 (6). This lineage is now the predominant lineage in Europe, with cases reported in Western, Central, Southern, and Southeastern Europe, while lineage 1 has primarily been described in southwestern Spain and Italy (7–13). Other lineages (3, 4, 7, 8, and 9) have also been detected in Europe, although they have not been connected with major outbreaks (6). The risk of introducing novel exotic lineages of WNV into Europe is primarily associated with migratory birds transiting through the Palaearctic-African flyway (14). In contrast, the local spread of the virus is not influenced by bird migration but relies on local transmission through mosquitoes (6, 14).

The first case of WNV in Serbia was recorded in 2010 when viral nucleic acid was detected in a mosquito pool, while the first case in birds was detected in 2012 from samples of both live and dead birds (12). Since then the number of cases has increased each year and reached its peak in 2018 with 415 human cases and 36 fatalities (12), while in 2022, the second-highest peak with 226 human cases was recorded (15). Additionally, a high seroprevalence of WNV has been detected in wild animals, highlighting an important but often overlooked circulation pathway of the virus (16). Considering the threat posed by WNV, an integrated government surveillance program is in place. This program aims to detect WNV nucleic acid in birds and mosquitoes, along with conducting serological surveillance of horses and calves as an early warning system. Since WNV has been circulating in Serbia for over a decade and is prone to frequent mutations, it is crucial to monitor its genetic evolution to understand its epidemiology and potential impacts on public health. In this study, we aimed to assess the phylogenetic relationships between previously sequenced strains from the country and those sequenced in this study. Additionally, we sought to identify possible virulence factors in Serbian strains that could influence the virus’s transmission dynamics, pathogenicity, and the effectiveness of control measures. By comparing the genetic makeup of different strains, we can gain insights into the evolutionary pressures acting on the virus and track the emergence of any new, potentially more virulent variants.

## 2 Materials and methods

### 2.1 Samples

The samples used in this study are from the Institute of Veterinary Medicine’s sample bank. Originally the samples were collected as a part of the West Nile virus surveillance program, funded by the Veterinary Directorate of the Ministry of Agriculture, Forestry and Water Management and included samples from mosquitos and tracheal swabs from birds. In the study, only samples previously characterized as positive by real-time qPCR with a Ct value below 25 were used for sequencing. In total 12 samples were selected, 1 from a mosquito, and 11 from birds (Supplementary material 1).

### 2.2 Extraction of nucleic acid and real-time qPCR detection

The viral nucleic acid was isolated using the IndiSpin Pathogen Kit (Indical Bioscience GmbH, Leipzig, Germany) following the instructions provided by the manufacturer. To confirm the successful extraction of nucleic acid, each sample included an external VetMax Xeno Internal Positive RNA Control (Applied Biosystems, Thermo Fisher Scientific, Massachusetts, USA). For the detection of nucleic acid of WNV, the following master mix for rtRT-PCR was prepared: 10 μL of Luna Universal Probe One-Step Reaction Mix (New England BioLabs, Ipswich, MA, USA), 0.8 μL of each 10 mM primer, 0.4 μL of 10 mM probe, 1 μL of RT Enzyme Mix, 5 μL of template, and 1 μL of Xeno Internal Positive RNA Control Primer. The remaining volume, up to 20 μL, was filled with nuclease-free water (RT-PCR Grade Water, Thermo Fisher Scientific, Massachusetts, USA). The primers and probe used were previously published by Eiden et al. (17). The thermal profile used was programmed on the QIAquant 96 5plex (Qiagen, Les Ulis, France), according to the manufacturer’s instructions.

### 2.3 WNV amplification

Whole genome sequencing was done by utilizing the previously published targeted approach for amplicon sequencing of the WNV (18). Sequencing was performed on a MinION (Mk1C device) sequencing platform. The approach used can be divided into two major steps.

The first step involved first-strand cDNA synthesis and amplification using the following procedures: Initially, the cDNA synthesis mix was prepared, which included 1 μL of 50 ng/μl Random Hexamers, 1 μL of 10 mM dNTPs mix (both from the SuperScript™ III First-Strand Synthesis System, Invitrogen - Thermo Fisher Scientific, Waltham, Massachusetts, USA), and 11 μL of 1 μg/mL viral RNA. This mixture was incubated at 65°C for 5 min, then quickly transferred to −80°C for 1 min.

For the cDNA amplification step, the mix included the following components: 4 μL of 5X SSIV Buffer, 1 μL of 100 mM DTT, 1 μL of 200 U/μl SuperScript™ IV Reverse Transcriptase (all from the SuperScript™ IV Reverse Transcriptase kit, Invitrogen - Thermo Fisher Scientific, Waltham, Massachusetts, USA), and 1 μL of RNase OUT Recombinant RNase Inhibitor (Invitrogen - Thermo Fisher Scientific, Waltham, Massachusetts, USA). The cDNA synthesis mix (with viral RNA) and the cDNA amplification mix were combined in a single tube, resulting in a total volume of 20 μL. This mixture was then subjected to the following thermal profile: 42°C for 50 min, followed by 70°C for 10 min. The resulting cDNA was stored at −80°C for future use.

The second step involved the primer-specific amplification of WNV cDNA. Previously published primers were used for this step (18). The primers were divided into two pools (odd and even primers), each with a volume of 10 μL and a concentration of 100 μM. These pools were further diluted in a 1:10 ratio to achieve a final concentration of 10 μM. Each sample was then amplified in two parallel reactions, each containing a different primer pool in the following mix: 12.5 μL of Q5 High-Fidelity 2X Master Mix (New England Biolabs, Ipswich, MA, USA), 0.77 μL of each primer pool, 2.5 μL of cDNA, and PCR-grade water to a final volume of 25 μL. The thermal profile used for amplification included: initial activation at 98°C for 30 s, followed by 35 cycles of 98°C for 15 s, and annealing at 65°C for 5 min. The amplified pools from each step were then combined in a single tube.

### 2.4 Sample barcoding

The sample concentration was optimized to 200 fmol. The amount of DNA was measured by using a Qubit dsDNA HS (High Sensitivity) Assay Kit on a Qubit™ 4 device (Thermo Fisher Scientific, Massachusetts, USA) as per the manufacturers’ instructions. Samples with excessive amounts of DNA were diluted with RNA pure water. Barcoding of the samples was performed using the Native Barcoding Kit 24 V14 (SQK-NBD114.24) from Oxford Nanopore, UK, following the Ligation Sequencing Amplicons protocol with v14 chemistry. The prepared library was then loaded onto a 10.4.1 flow cell and sequenced using the MinION Mk1C device. Sequencing lasted for 12 h, with basecalling conducted using Guppy v. 24.02.16 in the MinKNOW GUI v. 5.9.18, and an initial quality score set to 9.

### 2.5 Bioinformatics analysis

Demultiplexing was performed using MinKNOW GUI v. 5.9.18. The additional quality check was performed with Seqkit v2.8.2^1^, and the primers were removed from the raw sequences using the iVar trim option (iVar v1.4.2)^2^. The resulting reads were mapped to the reference genome with Minimap2 v2.24-r1122^3^. The files generated were subsequently checked and indexed using Samtools 1.19.2^4^. Finally, consensus sequences were created with iVar v1.4.2 (see text footnote 2). The resulting consensus sequences were annotated using Geneious Prime (Dotmatics, Boston, MA, USA) and submitted to the GenBank NCBI.

### 2.6 Sequence and phylogenetic analysis

The sequences were aligned using the MAFFT alignment algorithm with 205 WGS (Supplementary material 1) from the GenBank (NCBI) and analyzed with Geneious Prime (Dotmatics, Boston, MA, USA) software. The aligned sequences were then used for phylogenetic analysis in the Molecular Evolutionary Genetics Analysis (MEGA X) software (19). The Maximum Likelihood method and Tamura Nei model employed in the study were selected based on the results from the “Find Best DNA/Protein Models” feature in MEGA X. Previously described nomenclature for WNV was used for classification of strains (20).

Virulence factors were assessed in both structural and nonstructural proteins as was previously described (2).

In the structural proteins, the analysis focused on the 15–17 N-linked glycosylation motif in the prM protein, the 154–156 glycosylation motif, and the E159 mutation in the E protein.

For the nonstructural proteins:

- NS1 Protein: Glycosylation sites were analyzed using the N-X-S/T motif via the NetNglyc server^5^. Additionally, the presence of the E154S mutation was evaluated.
- NS3 Protein: The 249th position was evaluated for the presence of proline, which is associated with virulence.
- NS4B Protein: The conserved positions D35, P38, W42, and Y45, as well as the C102 and E249th positions, were evaluated for their connection to virulent strains.
- NS5 Protein: The K at 61st, 128th, 218th, and 804th positions were assessed for the presence of virulence factors.

### 2.7 E protein homology model

**Figure 1:**
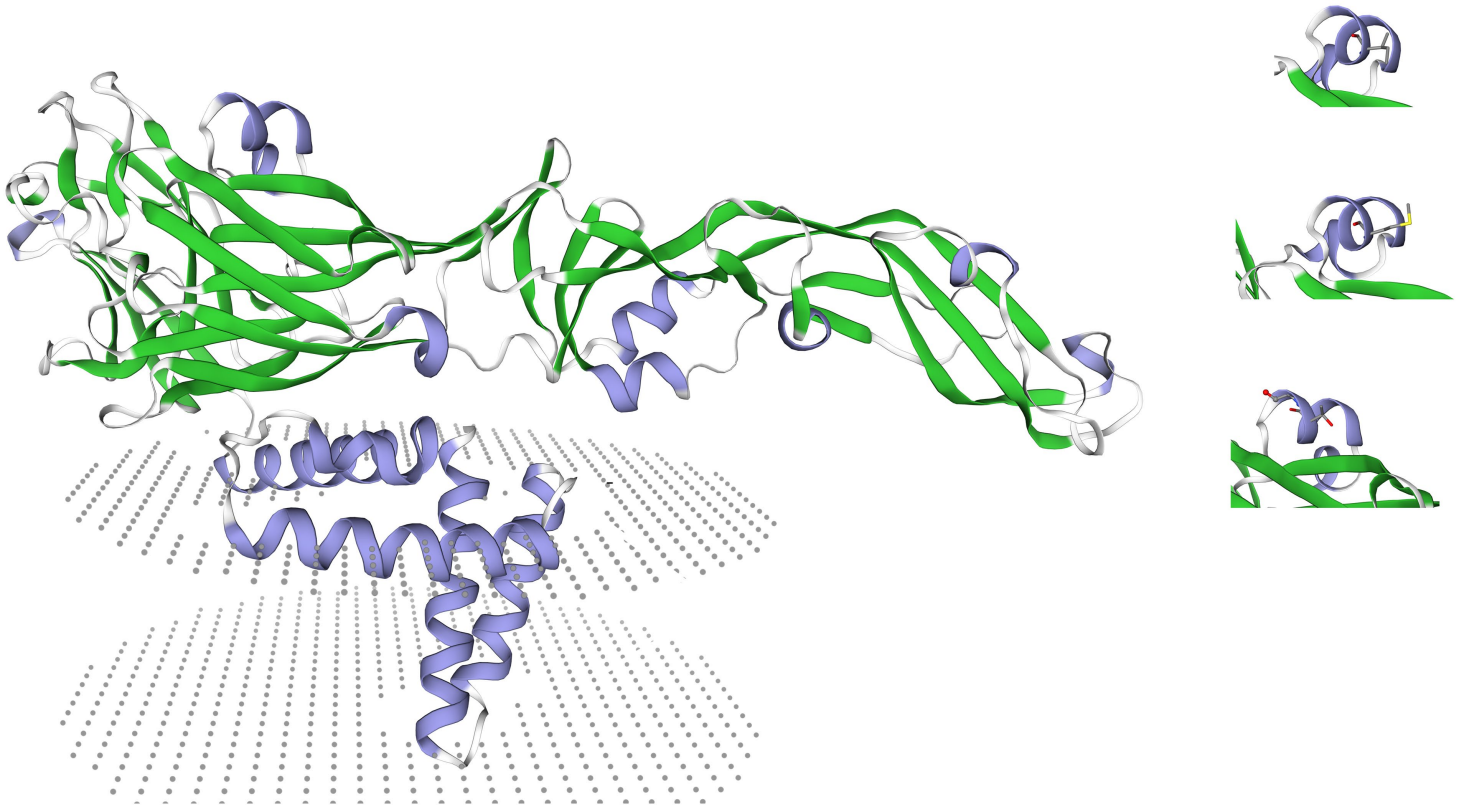
A presentation of a 3D homology model of the E protein created using the SWISS-MODEL online tool (https://swissmodel.expasy.org/). Labeled on the right side of the figure are the three aa changes at the E159th position, while on the left side, the entire E protein can be seen with the labeled glycolisation site and the transmembrane domain.

The molecular structure of the E protein was evaluated by creating a homology three-dimensional model using the SWISS-MODEL online tool^6^. To assess potential structural changes caused by mutations at the E159th position, three models were created with a focus on the E159th site (Figure 1).

Based on the phylogenetic trees obtained, a map of Europe was created using QGIS. Representatives from each sublineage were selected for mapping based on publicly available geolocation data from NCBI. Sequences that only listed the country of origin without specific location details were excluded. The coordinates for these representatives were approximated using the publicly available website Maps.ie^7^. It is important to note that the obtained coordinates represent the approximate locations of the sequenced strains.

## 3 Results

Sequencing was terminated after 12 h. The average quality score of 1,8 million generated reads was 14.78, the average base length was 552 bp, and the average sequencing depth was 4,300x. Out of 12 sequenced samples, good-quality sequences were obtained from 10 (83.3%). Four (33.3%) strains including SRB/WNF/6569/Belgrade 2018, SRB/WNF/8505-3/Belgrade 2018, SRB/WNF/8505/Belgrade 2018, SRB/WNF/8805-58846/Belgrade 2018 had gaps from the 8,909–9,505 nucleotide position, while the strain SRB/WNF/8805-58846/Belgrade 2018 had additional gaps in the 5,715–6,035 and the 6,877–7,395 positions. Excluding these 4 strains, the obtained genome length was 10,305 bp. High-quality sequences were processed and submitted to GenBank (NCBI) using the following accession numbers PQ053322 - PQ053331. Based on the BLAST similarity results the highest percent identity was recorded with Lineage 2 sequences PP212881.1 and PP212882.1 (Hungary) with 99.49 and 99.51%, respectively.

### 3.1 Phylogenetic analysis

**Figure 2:**
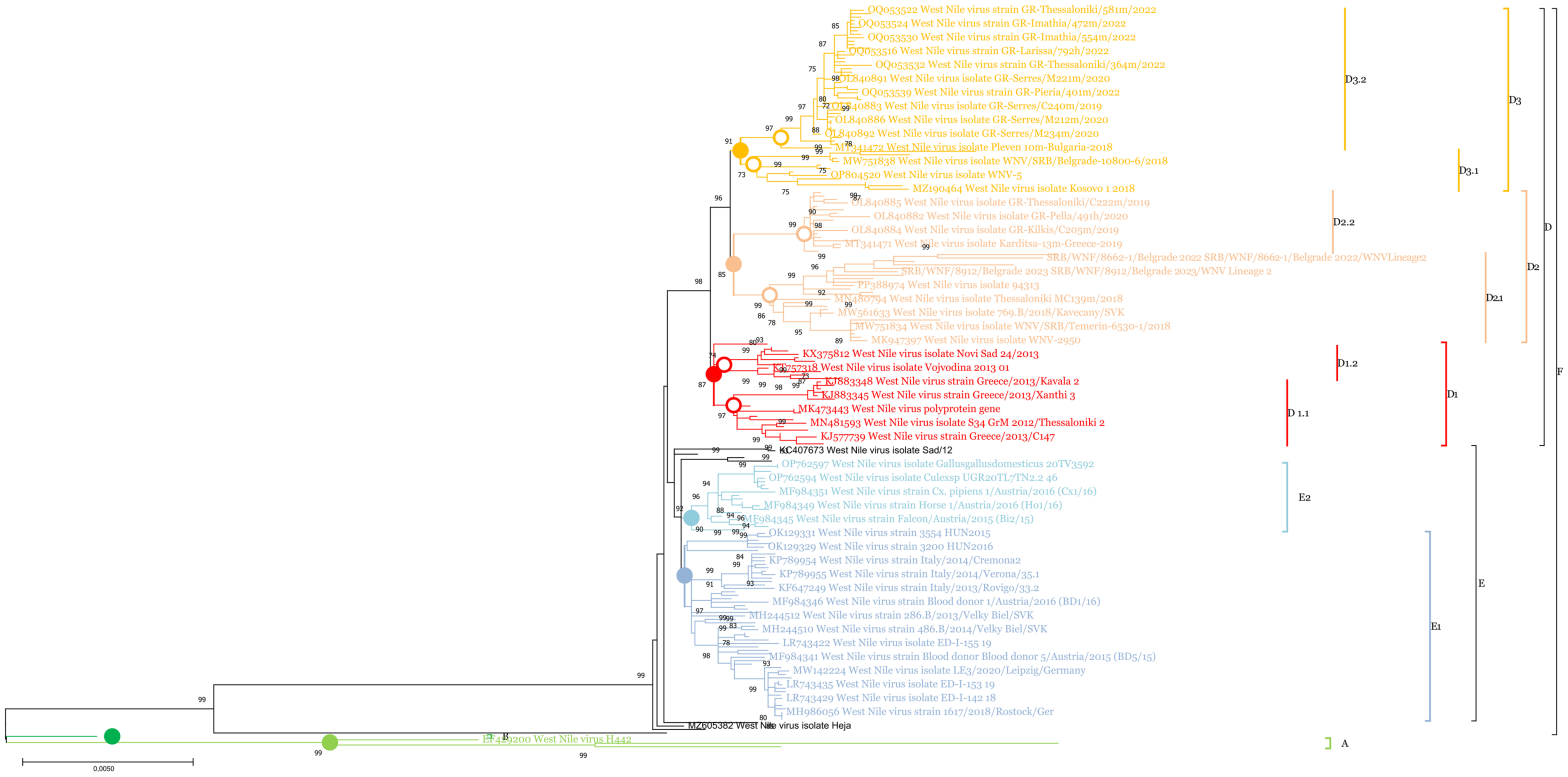
A condensed phylogenetic tree showing the genetic relationships between Serbian strains (PQ053322-PQ053331) and strains from the NCBI. Utilizing MEGA X software, the study employed the Maximum Likelihood Method alongside the Tamura Nei model for its investigation. To increase the accuracy 1,000 bootstrap replicates were included, a Gamma distribution to account for varying rates across sites (+G) across five rate categories, and the assumption that certain sites remain unchanged over time (+I). Branches not supported in at least 70% of the bootstrap replicates were condensed. To identify the most suitable model for analysis, the software’s “Find best DNA/Protein Models” functionality was utilized. Clades, and sublineages were labeled with the following colors with HTML color notations in brackets: Clade A light green (#91cf4f), Clade B dark green (#00b04f), sublineage E1 blue (#94b2d6), sublineage E2 light blue (#91ccdb), sublineage D1 red (#ff0000), sublineage D2 peach (#fabf8f), sublineage D3 orange (#ffbf00).

**Figure 3:**
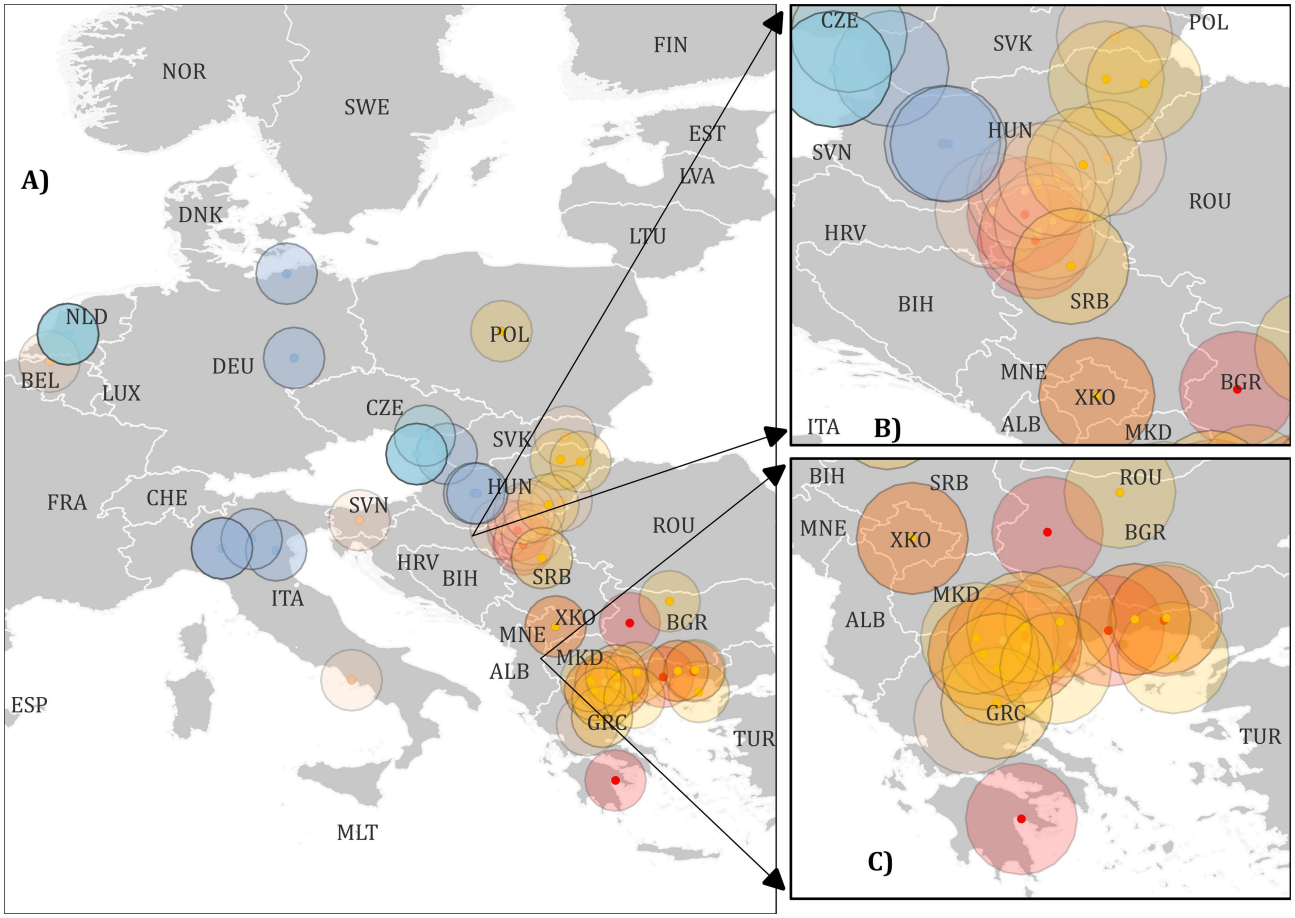
A map of Europe showing labelled locations of West Nile Virus (WNV) strains representing various sublineages used in the study. The dots and circles are color-coded as per the scheme below. Label A) indicates all European strains, label B) represents strains from Central Europe and the Balkans, and label C) represents strains from Southern Europe. Clades, and sublineages were labelled with the following colours with HTML colour notations in brackets: Clade A light green (#91cf4f), Clade B dark green (#00b04f), sublineage E1 blue (#94b2d6), sublineage E2 light blue (#91ccdb), sublineage D1 red (#ff0000), sublineage D2 peach (#fabf8f), sublineage D3 orange (#ffbf00).

Phylogenetic analysis revealed the existence of 4 major clades within WNV lineage 2 (A, B, C, and F). Clade F can be considered the European strain clade and was further divided into 2 subclades: E and D (Figure 2 and Supplementary Image 1). Clade E consisted of Western and Central European strains and was divided into two sublineages: E1 (Western-Central European strains from 2010 to 2018) and E2 (Western-Central European strains from 2015 to 2018). Within the E sublineage, 2 strains, MH244513 (West Nile strain 200.B/2013/Secovce/SVK) and OK129333 (West Nile strain 4,693 HUN2017), formed a distinct branch. Clade D comprised Central European, Western Balkan, and Southeast European strains, and was further divided into three sublineages: D1, D2, and D3. Sublineage D1 includes strains from the Western Balkans and Southeast Europe from 2010 to 2013, along with an additional strain, KC496015 (West Nile strain 578/10). These were further divided into D1.1 and D1.2. Sublineage D2 was further subdivided into 2 Southeast Europe sub-sublineages: D2.1, which includes Western Balkan strains from 2018 to 2023, and D2.2, which includes Southeast Europe strains from 2018. Sublineage D3 encompassed both Western Balkan and Southeast European strains, with the D3.1 sub-sublineage comprising strains from 2018 to 2019, while the D3.2 clade consisted entirely of Southeast European strains (Figure 3. Map). The circles were created to illustrate the potential overlapping of different strains.

### 3.2 Nonsynonimus mutation analysis

Of the 122 recorded aa changes, 30 were unique to our sequenced samples. Recorded changes in the sequenced samples are in Supplementary material 1.

No changes were observed in the N-linked 15–17 glycosylation site of the prM protein, nor at the M-36 or M-43 residues. In the E protein, the NYS motif at the 154–156 glycosylation site is preserved, while an EI159T mutation was evident in all sequenced strains. Additionally, no changes were detected in the 107 virulence motif across the sequenced strains. The E154S change was not observed in the sequenced samples. Based on the E protein homology model no changes in the folding of the three domains in the E protein were observed, besides the changes in the side chains of the aa at the E159th position.

For the NS1 nonstructural protein, glycosylation analysis revealed preserved N-glycosylation sites (NNTF/NTTE/NDTW) at the 135, 175, and 207 positions in all sequenced strains, except for the SRB/WNF/8870-10/Belgrade 2022/WNV Lineage 2 (PQ053330) strain, which had preserved glycosylation sites only at the 135th and 175th positions. Additionally, this strain showed changes at the 208th and 205th positions, specifically the D208G and T209P mutations. In the SRB/WNF/8805-58846/Belgrade 2018/WNV Lineage 2 (PQ053328) strain, the 207 glycosylation site experienced a change at the 208th position, with an NS1 D208H mutation, but the glycosylation site remained preserved (Supplementary material 1).

For the Serine protease/Helicase NS3 protein, proline was present at the 249th position, which has been described in highly virulent strains.

For the NS4B protein, no changes were observed at the D35, P38, W42, Y45, 102nd, or 249th positions.

For the NS5 protein, no changes were observed at the K61, K128, or K804 positions. However, a K218G mutation was observed at the 218th position in the PQ053331 strain, while no changes were observed in other strains.

## 4 Discussion

In this study, we report on the phylogenetic and molecular changes observed in strains sequenced directly from clinical samples, following a previously published protocol (18). Whole genome sequencing provides deeper insights into the evolution of WNV and valuable data for molecular epidemiology. Srihi et al. (20) described the existence of six different clades (A-F) within lineage 2 of WNV, with clades A, B and C being of primarily African strains. In Europe, there were two active subclades, D and E, which were also labeled as the southeastern European and Central/Eastern European clades, respectively (20).

In our study, we identified two major sublineages within the E subclade, designated as E1 and E2. These sublineages represented the majority of strains from Western and Central Europe between 2013 and 2018. Notably, three strains have separated from the others: WNV/SRB/Curug-5871/2018 (KC407673) and West Nile virus isolate Novi Sad/12 (KF179640) from Serbia, dated 2018 and 2012 respectively, and the West Nile virus strain Austria/2008 gh from an Austrian goshawk (KF179640). The earliest detection of strains from this E subclade can be traced back to 2008 in Austria, as well as strains from Serbia and Hungary from 2012. These early detections may represent the origin of these sublineages.

Sublineage D1 comprises strains from 2010 to 2015, originating from Greece and the Western Balkans. Our analysis indicated that sublineage D1 was no longer dominant in this region, suggesting other sublineages have supplanted it. Sub-sublineage D2.1, which includes strains from Greece dated 2018–2019, may represent a localized and self-limiting circulation of this sub-sublineage. In contrast, sub-sublineage D2.2 includes strains from the Western Balkans, Hungary, and a single strain from Italy, all collected between 2018 and 2023. Additionally, this sub-sublineage includes a strain from Belgium, identified in a previous study by Srihi et al. (20), which represents the earliest instance of this sub-sublineage, dating back to 2017. The majority of the strains sequenced in this study belong to sub-sublineage D2.2, suggesting its ongoing circulation in the Western Balkan region and potential for further spread, as evidenced by a strain from Italy identified in the fall of 2023. Sub-sublineage D3.1 includes strains from Western Balkan countries, Hungary, a single strain from Greece dated 2018–2019, and a strain from Poland dated to 2022. On the other hand, sub-sublineage D3.2 consists exclusively of strains from Greece, spanning from 2018 to 2022. This pattern suggests that D3.2 may represent a currently circulating sublineage within Greece.

The highest diversity of sublineages can be observed in southern Greece and the Western Balkans, where three different sublineages have circulated and overlapped since 2010. This study highlights that the Western Balkan and Central European countries are important mixing grounds for both Western-Central European strains (Clade E) and Southern European strains (Clade D). This finding aligns with the proposal by Srihi et al. (20), which identified Austria and Hungary as significant dissemination centers for WNV in Europe. This is further evidenced by the detection of strains from these sublineages in more northern locations, such as the D2.2 strain West Nile virus isolate WNV/Belgium/2017/Antwerpen (MH021189) from Belgium and the D3.1 strain West Nile virus isolate WNV-5 (OP804520.1) from Poland. These findings underscore the widespread dissemination and the complex epidemiology of WNV in Europe.

Protein characterization revealed no aa differences in the Capsid protein C, PROPEP, and Protein prM genomic regions in sequences from Serbia.

Virulence factors for WNV have been previously described, with major factors found primarily in the E protein. Variations in virulence have previously been linked to changes in the E protein particularly at the E154-156 (NYS) glycosylation site and the EI159V aa change. Based on the study by Davis et al. (21) the EV159 strains have been described as more virulent. A later study by Kobayashi et al. (5) suggested that the E159 site plays a crucial role in modulating pathogenicity, viral replication, and T-cell recruitment. The E154-156 glycosylation site remains preserved in our sequenced samples, while the E159th position exhibits an EI159T aa change. Chronologically, Serbian strains from 2010 initially showed an EI159M mutation at this site. Over time, these strains were replaced by those containing isoleucine at the E159th position. However, starting in 2018, both the EI159 and EI159T strains began circulating simultaneously. By 2022, the EI159T became the only observed change at this position in our samples, suggesting that it may now be the dominant strain in the region. Notably, this same EI159T aa change has also been recorded in the majority of strains from Kosovo and the Vojvodina autonomous region in 2018. A similar strain shift was observed in Austria when other regional strains were examined. Strains with isoleucine at the E159 position were detected in 2008, while strains from 2014 to 2015 predominantly show the EI159T change. This pattern was also seen in sequences from Greece. Strains from 2010 to 2013 primarily exhibit the EI159 aa change, but this is later replaced by the EI159T in samples sequenced after 2020. Given the importance of the E159 site, frequent changes in its aa sequence warrant further investigation. Isoleucine is a branched, highly hydrophobic aa, similar to valine and methionine, indicating that the EI159V and EI159M changes might not significantly affect peptide stability. In contrast, threonine (EI159T) is a polar aa with a hydrophilic side chain, which may lead to differences in protein folding or stability. This is particularly important considering that these mutations are adjacent to the E154-156 glycosylation sites.

The NS1 glycoprotein plays a crucial immunomodulatory role, enabling the virus to influence the activity of the complement system. In Flaviviruses, the NS1 glycoprotein typically features a conserved glycosylation motif (N-X-S/T) at 2 positions in Dengue Virus (DFV), Japanese Encephalitis Virus (JEV), and Yellow Fever Virus (YFV) (22). However, in the case of WNV, there are three such glycosylation motifs. In our study, we identified two strains with changes in the conserved region of the NS1 gene. The SRB/WNF/8870-10/Belgrade 2022/WNV Lineage 2 (PQ053330) strain exhibited two changes: D208G and T209P within the glycosylation site. According to Whiteman et al. (22), WNV mutants with ablated glycosylation sites showed decreased virulence in *in-vivo* mouse models. The reduction in virulence was correlated with the number of glycosylation sites ablated (22). The SRB/WNF/8805-58846/Belgrade 2018/WNV Lineage 2 (PQ053328) strain exhibited a D208H aa change at the second position of the third glycosylation site. Although the glycosylation site was recognized by the NetNglyc server, the substitution of aspartic acid with histidine could disrupt the protein’s secondary or tertiary structure. Aspartic acid is negatively charged, while histidine is positively charged, which might lead to protein misfolding. Previous studies have shown that any changes in glycosylation sites can affect the virus’s interaction with the immune system, potentially impairing the NS1 protein’s immunomodulatory role, thus resulting in attenuated WNV phenotypes (22, 23).

The NS3 protein, in conjunction with the NS2B protein, is involved in cleaving junctions between non-structural proteins that comprise the WNV polyprotein (24). Along with NS5, NS3 is essential for viral replication, and the creation of the viral replication complex (24). The NS3 protein spans all three domains and contains the RNA helicase and RNA triphosphatase activities at its C-terminal end. In a study by Brault et al. (25), a proline residue at the 249th position was identified as a virulence factor in a strain isolated from American crows, which exhibited high mortality rates. The proline residue at the 249th position was identified in all strains sequenced in our study. The same residue was found in previously sequenced strains from Serbia, except for 4 strains: West Nile virus strain Novi Sad 2010 (KC496016), West Nile virus strain Novi Sad/12 (KC496073), and 2 from 2018, WNV/SRB/Curug-5871/2018 (MW751837) and WNV/SRB/Subotica-6530-10/2018 (MW751846), where an NS3 P149H aa change was recorded. The virulent 249 proline residue was found in all strains from Greece and some strains from Hungary, while strains from central and Western Europe had the NS3 P249H aa change, which is in accordance with previously published studies (9).

A limitation of this study is the exclusion of environmental factors like climate change, which can affect mosquito populations and the spread of West Nile Virus. Future studies should include these factors for a more complete understanding of transmission dynamics. Additionally, implementing strong public health measures, such as mosquito control and public awareness programs, is crucial. Enhancing collaboration and data sharing across European countries would further improve the understanding of WNV patterns and support coordinated efforts to address outbreaks.

## 5 Conclusion

The study highlights that the Western Balkan and Central European countries are significant mixing grounds for WNV strains from both Western-Central Europe (Clade E) and Southern Europe (Clade D). The spread of these strains, including to Belgium and Poland, underscores the complexity of WNV dissemination in Europe. This is the first in-depth molecular analysis of the circulating WNV strains in Serbia. The study provides insights into the evolution of WNV in Serbia and Europe, noting the importance of continuous monitoring and phylogenetic analysis to track the emergence and spread of virulent strains. The frequent changes observed in critical sites like E159, the NS1 and the NS3 protein warrant further investigation to understand their impact on the virus’s pathogenicity and epidemiology.

## Data Availability

The datasets presented in this study can be found in GenBank NCBI. The accession numbers: PQ053322: https://www.ncbi.nlm.nih.gov/nuccore/PQ053322, PQ053323: https://www.ncbi.nlm.nih.gov/nuccore/PQ053323, PQ053324: https://www.ncbi.nlm.nih.gov/nuccore/PQ053324, PQ053325: https://www.ncbi.nlm.nih.gov/nuccore/PQ053325, PQ053326: https://www.ncbi.nlm.nih.gov/nuccore/PQ053326, PQ053327: https://www.ncbi.nlm.nih.gov/nuccore/PQ053327, PQ053328: https://www.ncbi.nlm.nih.gov/nuccore/PQ053328, PQ053329: https://www.ncbi.nlm.nih.gov/nuccore/PQ053329, PQ053330: https://www.ncbi.nlm.nih.gov/nuccore/PQ053330, and PQ053331: https://www.ncbi.nlm.nih.gov/nuccore/PQ053331.

## Footnotes

^1^ https://bioinf.shenwei.me/seqkit/

^2^ https://github.com/andersen-lab/ivar

^3^ https://github.com/lh3/minimap2/releases

^4^ https://github.com/samtools/samtools/releases/

^5^ https://services.healthtech.dtu.dk/service.php?NetNGlyc-1.0

^6^ https://swissmodel.expasy.org/

^7^ https://www.maps.ie/coordinates.html

## Supplementary material

Supplementary Image 1 A complete phylogenetic tree showing the genetic relationships between Serbian strains (PQ053322-PQ053331) and strains from the NCBI. Utilizing MEGA X software, the study employed the Maximum Likelihood Method alongside the Tamura Nei model for its investigation. To increase the accuracy 1000 bootstrap replicates were included, a Gamma distribution to account for varying rates across sites (+G) across five rate categories, and the assumption that certain sites remain unchanged over time (+I). Branches not supported in at least 70% of the bootstrap replicates were condensed. The software’s “Find best DNA/Protein Models” functionality was utilized to identify the most suitable model for analysis.Clades and sublineages were labelled with the following colours with HTML colour notations in brackets: Clade A light green (#91cf4f), Clade B dark green (#00b04f), sublineage E1 blue (#94b2d6), sublineage E2 light blue (#91ccdb), sublineage D1 red (#ff0000), sublineage D2 peach (#fabf8f), sublineage D3 orange (#ffbf00).
